# Age specific fast breathing in under-five diarrheal children in an urban hospital: Acidosis or pneumonia?

**DOI:** 10.1371/journal.pone.0185414

**Published:** 2017-09-27

**Authors:** Sharika Nuzhat, Tahmeed Ahmed, Chowdhury Ali Kawser, Azharul Islam Khan, S. M. Rafiqul Islam, Lubaba Shahrin, K. M. Shahunja, Abu S. M. S. B. Shahid, Abdullah Al Imran, Mohammod Jobayer Chisti

**Affiliations:** 1 Nutrition and Clinical Services Division (NCSD), International Center for Diarrheal Disease Research, Bangladesh (icddr,b), Dhaka, Bangladesh; 2 Department of Pediatrics, Bangabandhu Sheikh Mujib Medical University (BSMMU), Dhaka, Bangladesh; Kliniken der Stadt Köln gGmbH, GERMANY

## Abstract

**Background:**

Children with diarrhea often present with fast breathing due to metabolic acidosis from dehydration. On the other hand, age specific fast breathing is the cornerstone for the diagnosis of pneumonia following classification of pneumonia recommended by the World Health Organization (WHO). Correction of metabolic acidosis by rehydrating the diarrheal children requires time, which delays early initiation of appropriate antimicrobials for pneumonia and thereby increases the risk of deaths. We need to further investigate the simple clinical features other than fast breathing which might help us in earliest diagnosis of pneumonia in children with diarrhea Thus, the objective of our study was to identify other contributing clinical features that may independently help for early diagnosis of pneumonia in diarrheal children who present with age specific fast breathing.

**Methods:**

This was an unmatched case-control study. Diarrheal children aged 0–59 months, admitted to Dhaka Hospital of the International Centre for Diarrheal Disease Research, Bangladesh (icddr,b) during January 2014 to December 2014 having age specific fast breathing (<2 month ≥60 breath/min, 2–11 months ≥50 breaths/min, >11–59 months ≥40 breaths/min) were studied. The study children with clinical and radiological pneumonia constituted the cases (n = 276) and those without pneumonia constituted the controls (n = 446). Comparison of clinical features and outcomes between the cases and the controls was made.

**Results:**

The distribution of acidosis among the cases and the controls was comparable (35% vs. 41%, p = 0.12). The cases had proportionately higher deaths compared to the controls, however, the difference was not statistically significant (3% vs. 1%; p = 0.23). In logistic regression analysis after adjusting for potential confounders, the cases were independently associated with cough (OR = 62.19, 95% CI = 27.79–139.19; p<0.01) and chest wall indrawing (OR = 31.05, 95%CI = 13.43–71.82; p<0.01) and less often had severe acute malnutrition (OR = 0.33, 95%CI = 0.13–0.79; p<0.01). The sensitivity and specificity of cough were 83% (78–87%) and 93% (91–96%). The sensitivity and specificity for lower chest wall indrawing were 65% (59–71%) and 95% (93–97%). However, the sensitivity and specificity of cough and lower chest wall indrawing combined were 94% (89–97%) and 99% (97–100%).

**Conclusion and significance:**

Thus, diarrheal children having fast breathing who present with cough and/or lower chest wall indrawing, irrespective of presence or absence of metabolic acidosis, are more likely to have radiological pneumonia. The results underscore the importance of early identification of these simple clinical features that may help to minimize potential delay due to rehydration in initiating prompt treatment of pneumonia in order to reduce fatal consequences in such children.

## Introduction

Pneumonia and diarrhea are the two major causes of deaths in children under five and accounted for about 16% and 9% deaths respectively among the 5·8 million under-five global deaths in 2013 [1]. More than 95% of these deaths occur in developing countries especially in Sub-Saharan Africa and South Asia [1, 2]. In Bangladesh, among 119,000 annual deaths in children under-five in 2015, 15% were from pneumonia and 6% from diarrhea [1]. There is increased mortality among children with pneumonia who have co-morbid diarrhea [3]. Diagnosis followed by early management of these children is critically important to reduce the morbidity and mortality.

Children with diarrhea often present with fast breathing due to metabolic acidosis from dehydration and/or sepsis [4–7]. On the other hand, age specific fast breathing is the cornerstone for the diagnosis of pneumonia following classification of pneumonia recommended by the World Health Organization (WHO) [8]. Clinicians often wait until full correction of dehydration that used to help in correcting metabolic acidosis and simultaneously reassess whether fast breathing still persists after rehydration. Presence of fast breathing after correction of dehydration helps the clinicians in diagnosing pneumonia in children with diarrhea. However, correction of metabolic acidosis by rehydrating the diarrheal children requires time [9–11], which delays early initiation of appropriate antimicrobials required for the treatment pneumonia and thereby increases the risk of deaths [12]. Moreover, children with diarrhea may also have metabolic acidosis in absence of dehydration especially in sepsis, although, this special group of children also require prompt antibiotics. Thus, further evaluation of simple clinical features that might help clinicians in earliest diagnosis of pneumonia is warranted to initiate antibiotic treatment promptly. However, clinical studies looking for the role of fast breathing in differentiating pneumonia from metabolic acidosis in children with diarrhea are limited [7, 13]. Additionally, there is often lack in availability of laboratory investigation for the diagnosis of acidosis in resource poor setting. Thus, the purpose of our study was to identify other contributing clinical features that may independently help for early diagnosis of pneumonia in diarrheal children who present with age specific fast breathing.

## Methods

### Ethics statement

In this retrospective chart analysis, data were de-identified before analysis and hence no parental consent was required. The Ethical Review Committee, an institutional review board of the International centre for diarrheal disease research, Bangladesh (icddr,b), reviewed and approved the study before the study began.

### Study design

This was a retrospective chart analysis that was conducted at Dhaka Hospital of icddr,b using electronic database of the hospital. We used an unmatched case control design and enrolled diarrheal children of both sexes, aged 0–59 months, with age specific fast breathing who were admitted to the Longer Stay Ward (LSW) that includes Acute Respiratory Illness (ARI) ward and Intensive Care Unit (ICU) of the Hospital, from January 2014 to December 2014.Children with fast breathing presenting with diarrhea and both clinical and radiological pneumonia constituted the ‘cases’ and diarrheal children without radiological pneumonia constituted ‘controls’.

Clinical pneumonia was diagnosed after full correction of dehydration or in absence of dehydration and the diagnosed was made following WHO classification of pneumonia [14]. Radiological pneumonia was also defined following WHO classification [15], and the diagnosis was made with the agreement of consultant and radiologist. Age specific fast breathing was defined if there was ≥60 breaths/min in children <2 months of age, ≥50 breaths/min in 2–11 months, and ≥40breaths/min in >11–59 months. Severe acute malnutrition (SAM) was defined if weight for length/height Z score in a child was <-3 or had nutritional edema [8]. Diarrhea was defined if there is passage of three or more abnormally loose or watery stools per 24 hours, and status of dehydration was defined by “Dhaka Methods” of assessment of dehydration that is almost similar to WHO method and has been approved by the WHO [10].

### Study site

Dhaka hospital of icddr,b, each year provides care and treatment of 140,000 patients of all age groups. The majority of the patients come from poor socio-economic background, living in urban and peri-urban Dhaka. This being mainly diarrhea treatment facility, essentially all patients have diarrhea with or without associated complications. More than 60% patients are under-five children. Malnutrition and pneumonia are the most common co-morbidities. Other description of study site has been provided elsewhere [16].

### Patient management

On arrival to the hospital the triage nurses obtain medical history and perform physical examination and make quick assessment of dehydration status. The nurses also evaluate the complications and other health problems associated with diarrhea, particularly malnutrition and pneumonia. The patients are then referred to the emergency physicians who re-asses and admit them to an appropriate ward of the hospital. After the admission the respective ward physicians further re-evaluate the patients, initiate the needed works up and prescribe management according to protocols those are followed in the Dhaka hospital of icddr,b and ensure its implementation. Management of the patients following hospital protocols has also been described elsewhere [16].

### Measurements

For relevant Data acquisition case report forms (CRFs) were developed and finalized. The key outcome variables those were analyzed include fever, cough, chest wall indrawing, and dehydrating diarrhea (some or severe dehydration). Along with key outcome variables other major independent variables include demographic information (age, sex, socio-economic status), feeding status before 6 months of age, immunization status, nutritional status (expressed by age and sex specific ‘z’ score and classified as per WHO classification system) [17], type and dehydration status of watery diarrhea, clinical features of pneumonia defined by WHO [8], hypoxemia [arterial oxygen saturation (SPO2) <90% in room air] [8]. Congenital heart disease was clinically diagnosed and confirmed by echocardiography as recorded in the data set. Presence of metabolic acidosis (serum total carbon di-oxide level <18mmol/L) on admission and hospital acquired infection (clinical evidence of new infection after 48 hours of hospitalization and 72 hours after discharge from hospital stay) were also analyzed as confounder. Outcome of the patient was also recorded.

### Analysis

All data were entered into SPSS for Windows (version 20·0; SPSS Inc. Chicago) and Epi-Info (version 7·0, USD, Stone Mountain, GA). Difference in proportion were compared by the Chi—square test, student’s t-test was used to compare the means of normally distributed data and Mann-Whitney test was used for comparison of data that were not normally distributed. For statistical significance level α considered at level 0.05. Strength of association was determined by calculating odds ratio (OR) and their 95% confidence interval (CIs). In identifying predicting factors for pneumonia in children presenting with age specific fast breathing, variables were initially analyzed in a uni-variate model and then independently associated predictors were identified using logistic regression by backward stepwise method after controlling for the potential co-variates. The factors with p<0.05 having very low proportion (<1%) in controls were not put in logistic regression model as those were rejected by the model during the analysis. We further evaluated the sensitivity and specificity of the significantly associated clinical features with pneumonia and their 95% confidence intervals.

## Results

Among a total of 722 enrolled children 276 were cases, and 446 were controls. The cases more often presented with lower age, dehydration, fever, cough, hypoxemia, chest wall indrawing, reluctant to feed, grunting respiration and less often with SAM compared to the controls (Table 1). A total of 93 cases (35%) and 184 (41%) controls had acidosis on presentation, however, the difference was not statistically significant (p = 0.12). In logistic regression analysis the variables which had less than 1% value were removed (hypoxemia, reluctance to feed, grunting) as those were rejected by the logistic model. In logistic regression analysis after adjusting for potential confounders such as fever and dehydration, under five diarrheal children with pneumonia having age specific fast breathing more often had cough and chest wall indrawing, and less often had SAM compared to the controls (Table 2). The sensitivity and specificity of the significantly associated clinical features with pneumonia and their 95% confidence intervals have been shown in Table 3.

**Table 1:** Clinical characteristics of under-five diarrheal children with (cases) and without pneumonia (controls) on admission and their outcome.

Characteristics	Cases (n = 276) (%)	Controls (n = 446) (%)	OR	95% CI	p value
Male sex	187 (25.9%)	305 (42.2%)	0.97	0.69–1.36	0.92
Age in months (median, IQR)	8.0 (5.0,12.0)	13.0(6.0,19.0)	-	-	<0.01
Poor socio-economic condition	9 (3.3%)	4 (0.9%)	0.75	0.01–13.68	0.68
Exclusive breast feeding	59 (21.4%)	110 (24.7%)	0.89	0.60–1.33	0.20
Immunization as per EPI	159 (57.6%)	288 (64.6%)	1.13	0.72–1.80	0.65
Acute watery diarrhea	256 (92.8%)	416 (93.5%)	0.89	0.48–1.70	0.81
Dehydrating diarrhea	50 (18.1%)	141 (31.6%)	0.48	0.32–0.70	<0.01
Metabolic acidosis	97 (35.1%)	184 (41.3%)	0.77	0.56–1.06	0.20
Fever	206 (74.6%)	188 (42.2%)	8.59	5.59–13.48	<0.01
Cough	229 (83.0%)	29 (6.5%)	139.73	76.32–258.09	<0.01
Chest wall indrawing	180 (65.2%)	22 (4.9%)	64.24	36.52–114.65	<0.01
Hypoxemia	19 (6.5%)	0 (0.0%)	-	-	<0.01
Reluctance to feed	24 (8.7%)	3 (0.7%)	-	-	<0.01
Grunting respiration	8 (2.9%)	0 (0.0%)	-	-	<0.01
Nasal flaring	5 (1.8%)	0 (0.0%)	-	-	<0.01
Vomiting	213 (77.2%)	396 (88.8%)	0.92	0.55–1.57	0.85
Convulsion	12 (4.3%)	10 (2.2%)	1.98	0.77–5.18	0.17
Congenital heart disease	19 (6.9%)	14 (3.1%)	1.21	0.31–4.56	0.99
Severe acute malnutrition	56 (20.3%)	163 (36.5%)	0.45	0.31–0.65	<0.01
Sepsis	10 (3.6%)	9 (2.0%)	-	-	0.59
Hospital acquired infection	10 (3.6%)	16 (3.6%)	-	-	0.34
Death	8 (3%)	6 (1%)	2.19	0.66–7.72	0.23

Figures represent n (%), unless specified. OR: odds ratio. CI: confidence interval. IQR: inter-quartile range. EPI: Expanded Program on Immunization; SD: standard deviation;

**Table 2:** Results of logistic regression analysis to explore the independent predicting factors of pneumonia in diarrheal children having age specific fast breathing.

Characteristics	OR	95%CI	p value
Fever on admission	1.16	0.44–3.06	0.76
Cough on admission	62.19	27.78–139.19	<0.01
Dehydrating diarrhea	0.65	0.26–1.61	0.35
Lower chest wall in-drawing	31.05	13.43–71.82	<0.01
Severe acute malnutrition	0.33	0.13–0.79	0.014

**Table 3:** Sensitivity and specificity of the clinical predictors of pneumonia in diarrheal children having age specific fast breathing.

Variables	Sensitivity (95% CI)	Specificity (95% CI)
Cough	83 (78–87)	93 (91–96)
Lower chest wall indrawing	65 (59–71)	95 (93–97)
Hypoxemia	4 (3–6)	99 (98–100)
Reluctance to feed	9 (6–13)	99 (98–100)
Grunting respiration	3 (1–6)	99 (98–100)
Nasal flaring	2 (1–4)	99 (98–100)
Cough and lower chest wall indrawing combined	94 (89–97)	99 (97–100)

## Discussion

Early identification of pneumonia in children with diarrhea is troublesome and usually based on combination of history, clinical and radiological findings. The clinical diagnostic strategy for childhood pneumonia recommended by the WHO did not address dehydration and acidosis which can change respiratory rate [18]. Fast breathing and chest indrawing are the two cornerstone clinical signs of pneumonia that formed the basis of the WHO pneumonia case management strategy. Although the performance of these two clinical signs in diagnosing pneumonia in non-diarrheal children is reasonable, their performance is variable in diarrheal children [4, 7, 13]. However, none of these studies [4, 7, 13] had evaluated other clinical features of pneumonia in diarrheal children presenting with age specific fast breathing and to our knowledge this is the first study that evaluated the supportive clinical features with age specific fast breathing for the diagnosis of pneumonia in children with diarrhea.

The most important observation of this study is the case-mix of acidosis and pneumonia in diarrheal children who present with fast breathing. Another important observation is the independent association of additional clinical feature in diagnosing pneumonia in such children.

Our observation of cough and lower chest wall indrawing as the independent predictors of pneumonia in diarrheal children having fast breathing irrespective of presence or absence of metabolic acidosis is very important for the clinicians in developing countries. Because there is lack of availability of biomedical tools in diagnosing metabolic acidosis and access to chest X-ray is often limited in resource-poor settings. Although sensitivity and specificity of cough and lower chest wall indrawing as an individual feature are good, 94% sensitivity and 99% specificity of cough and lower chest wall indrawing combined bolster the strong predictive value of these clinical features combined in diagnosing pneumonia in this special population with high public health importance. This observation will help in minimizing the time lapse potentially for the correction of dehydration and metabolic acidosis in diarrheal children presenting with fast breathing and ultimately simplify the diagnosis of pneumonia in such children. The sensitivity and specificity of cough and lower chest wall indrawing in diagnosing pneumonia in non-diarrheal children varied [19]. The sensitivity and specificity were reasonably high for the diagnosis of pneumonia in well nourished children [20], although, these values were found to be inconsistent in diagnosing pneumonia in malnourished children [19, 21]. However, this is the only study where independent or combined performance of cough or lower chest wall indrawing in diagnosing pneumonia in children with diarrhea has been well validated.

Our observation of association of hypoxemia, reluctance to feed, grunting respiration, or nasal flaring with pneumonia in diarrheal children having fast breathing is also very important. Very low sensitivity of these clinical features might discourage our clinicians to use these features as diagnostic tools of pneumonia in such children. On the other hand, very high specificity of these clinical features suggest the presence of these features indicates a high probability of presence of pneumonia in such children and underscores the importance of imperative check of these clinical features that may help significant reduction in mortality of such children especially in resource poor settings. A number of previous studies also validated the role of hypoxemia [22], reluctance to feed [8], grunting respiration [8], or nasal flaring [8] for the diagnosis of pneumonia in non-diarrheal children.

Our observation of lesser proportion of SAM in study children with pneumonia compared to those without pneumonia is understandable and might be due to the fact that a good number of children with SAM usually do not present with fast breathing resulting from poor inflammatory response and presence of electrolyte imbalance especially hypokalemia. The poor sensitivity and specificity of fast breathing in diagnosing pneumonia in SAM children had already been reported [17, 23].

The case-fatality-rate in children with pneumonia compared to those without pneumonia was proportionately higher, although, it was statistically insignificant. The important observation of this study is the overall lower deaths in this cohort of children with pneumonia compared to previous studies done in this hospital.[4, 23] The lower death rates in diarrheal children with pneumonia might be due to the fact that bubble CPAP has been incorporated as an essential part of standard of care for the management of children with severe pneumonia and hypoxemia in the Dhaka hospital of icddr,b since August 2103, just after the cessation of the randomized controlled clinical trial on bubble CPAP oxygen therapy using locally made ultra-low-cost bubble CPAP circuit, which had shown to have significant reduction in deaths in children with severe pneumonia and hypoxemia beyond the neonatal period compared to WHO standard low flow oxygen therapy.[16]

This study has a number of potentially important imitations. The main limitation of the study is the retrospective nature of the study, in identifying clinical predictors of pneumonia in children with diarrhea presenting with age specific fast breathing, prevented us from collecting information on a broader range of variables that may have been potential additional predictors. Another limitation is the lack of delineation of metabolic acidosis from respiratory acidosis as we have used the value of total carbon- dioxide from serum electrolytes report in defining acidosis in our study.

In conclusion, the results of our data suggest that diarrheal children who present with fast breathing additionally require of having history of cough and/or lower chest wall indrawing for the diagnosis of pneumonia in such children. However, hypoxemia, reluctance to feed, grunting respiration, or nasal flaring should also be looked for in maximizing the diagnosis of pneumonia and simultaneously in early initiation of antibiotics before completion of rehydration thereby may help in reducing deaths in such children especially in resource poor settings. A prospective study with more detailed evaluation of diarrhea with pneumonia is imperative to consolidate our observation.
